# miRNA regulation of Tip110 expression and self-renewal and differentiation of human CD34+ hematopoietic cells

**DOI:** 10.18632/oncotarget.23572

**Published:** 2017-12-21

**Authors:** Ying Liu, Xinxin Huang, Khalid A. Timani, Hal E. Broxmeyer, Johnny J. He

**Affiliations:** ^1^ Department of Microbiology, Immunology and Genetics, Graduate School of Biomedical Sciences, University of North Texas Health Science Center, Fort Worth, TX 76107, USA; ^2^ Department of Microbiology and Immunology, Indiana University, Indianapolis, IN 46202, USA

**Keywords:** hematopoiesis, CD34+, miRNA, 3′UTR, ceRNA

## Abstract

Tip110 expression regulates hematopoiesis, but the regulatory mechanisms during hematopoiesis are not fully understood. There are a number of putative microRNA (miRNA) binding sites identified within the Tip110 3′ untranslated region (3′UTR). In this study, we determined the relationship among Tip110 miRNA, Tip110 expression and self-renewal and differentiation of human CD34+ hematopoietic cells. Using a Tip110 3UTR-based reporter gene assay, 11 miRNA showed the specific activity toward the Tip110 3′UTR and down-regulated constitutive Tip110 mRNA expression. When human cord blood CD34+ cells were differentiated, Tip110 mRNA expression showed significant decreases. Concurrently, five miRNA showed significant increases, five miRNA showed significant decreases, and one miRNA remained unchanged. To further assess the roles of miRNA in Tip110 expression and self-renewal and differentiation of human CD34+ hematopoietic cells, human cord blood CD34+ cells were transduced to express the full-length Tip110 3′UTR RNA. Expression of the Tip110 3′UTR RNA led to significant increases of Tip110 mRNA, and the number of hematopoietic stem cells and progenitor cells. Taken together, these results show important roles of Tip110 miRNA in Tip110 expression control and Tip110 regulation of hematopoiesis and offer a possibility of using Tip110 miRNA or 3′UTR as a strategy to maintain human CD34+ hematopoietic cells.

## INTRODUCTION

Hematopoietic stem cells (HSCs) have the potential to self-renew for maintaining life-long hematopoiesis as well as for the production of multiple types of mature hematopoietic cells. It is important to understand how hematopoiesis is regulated in order to develop strategies for the enhanced production and use of HSCs. Understanding post-transcriptional regulation from genetic programs coming from intrinsic and extrinsic mechanisms will accelerate such progress.

MicroRNAs (miRNAs) are short non-coding RNAs and about 22 nucleotides in length, they regulate gene expression at a post-transcriptional level by inducing mRNA degradation and translational inhibition. Several miRNAs play a role in the regulation of hematopoietic cell differentiation [1]. How miRNAs regulate HSC self-renewal and progenitor cell lineage commitment is not well characterized. Examples of this regulation include miR-125a/b effects on HSC survival, in part through proapoptotic targets, Bmf and KLF13 [2–4]. miR-126 regulates HSC functions via the PI3K/AKT/GSK3β axis [5]. miR-29a has been implicated in early hematopoietic regulation [6]. Recently, we have reported that miR-124 specifically binds to Tip110 and regulates Tip110 expression followed by differentiation of human cord blood (CB) CD34+ cells, and production of hematopoietic progenitor cells (HPCs) *in vitro* [7].

Analyzing miRNA binding sites within the 3′UTR is important for understanding gene regulation. Many different RNA-binding proteins and miRNAs are predicted to interact with the 3′ untranslated region (3′UTR) Tip110 based on Tip110 sequences and structure [7]. Tip110 is an essential gene expressed in the earliest cells of adult bone marrow hematopoietic development. Tip110 protein is highly expressed in embryonic stem cells (ESCs) and HSCs, but is rapidly down-regulated following differentiation of these cells. Tip110 is important in regulation of HSC hematopoiesis through regulation c-myc [8, 9]. In addition, Tip110 is important in regulation of ESC through maintaining expression of pluripotent factors including NANOG, OCT-4, and SOX2 [10]. In this study, we were interested in understanding the relationship between Tip110 miRNA, Tip110 expression and self-renewal and differentiation of human CD34+ hematopoietic cells. We identified a group of miRNA that targeted Tip110 3′UTR and regulated Tip110 expression. We further demonstrated that ectopic expression of the Tip110 3′UTR RNA in human core blood CD34+ cells (enriched for HSCs and HPCs) led to increased constitutive Tip110 expression and the number of HSCs and HPCs during differentiation.

## RESULTS

### Tip110 3′UTR is targeted by more than 10 different miRNAs

**Figure 1:**
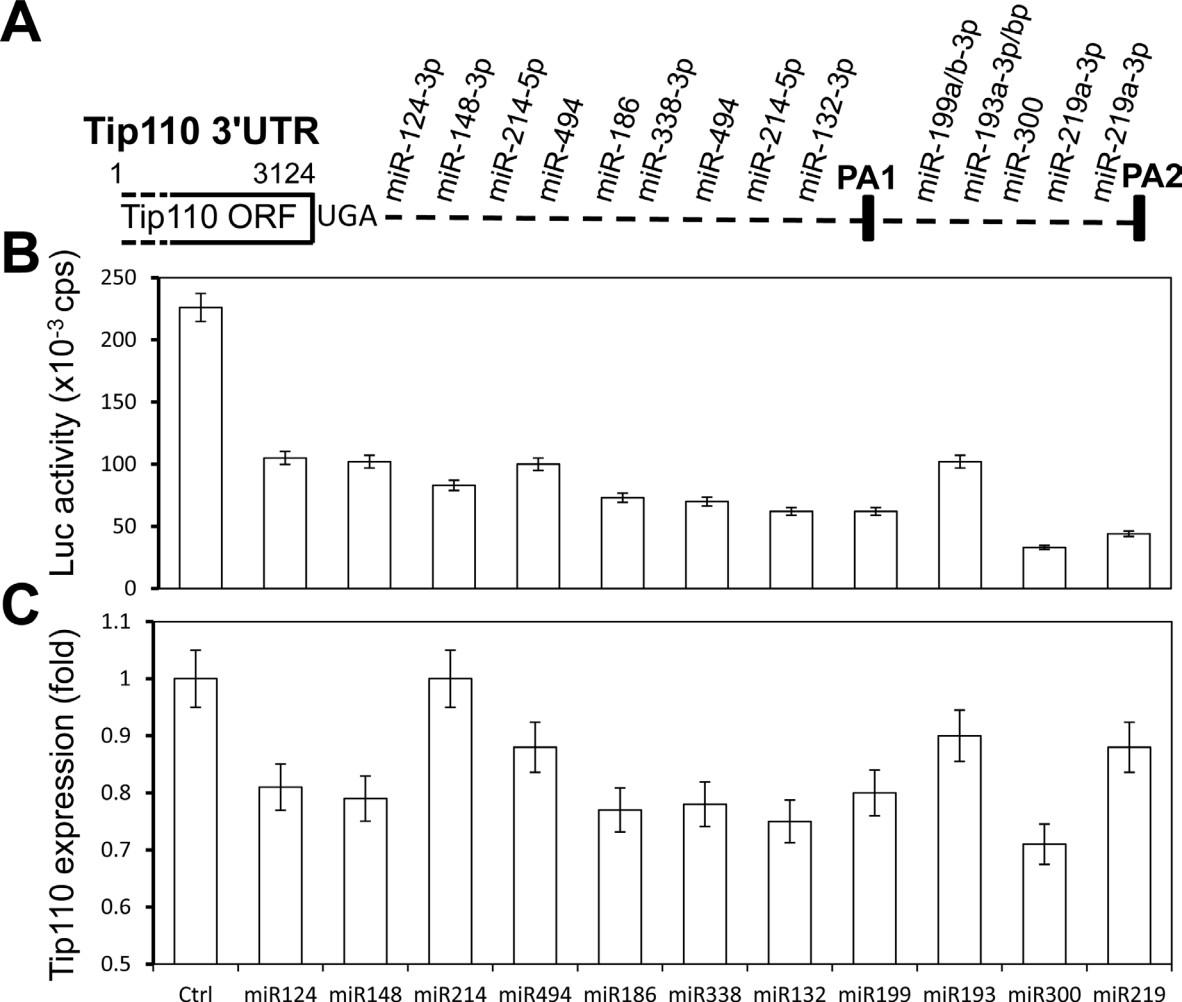
miRNAs specifically target the Tip110 3′UTR (**A**) Schematic of the Tip110 3′UTR region with predicted miRNA binding sites (Tip110 miRNA). (**B** and **C**) 293T were transfected with pLightSwitch-Tip110.3′UTR and miR-124, miR-148, miR-214, miR-494, miR-186, miR-338, miR-132, miR-199, miR-193, miR-330, or miR-219. Forty-eight hours after transfection, cells were harvested for cell lysates followed by the luciferase reporter assay (B) or for RNA isolation followed by qRT-PCR for Tip110 RNA expression (C). The fold changes of Tip110 mRNA expression were calculated using β-actin as the reference. pTK-βgal plasmid was included to normalize the transfection efficiency variations among all transfections. The data are the mean ± SEM of triplicates and are representative of three independent experiments.

We have previously shown that miR-124 down-regulates Tip110 expression by directly targeting its 3′ UTR [7]. We have also shown that Tip110 expression is important for hematopoiesis [8]. To determine whether there would be other potential miRNA binding sites within the Tip110 3′UTR, we used two popular programs (TargetScanHuman 6.2 and microRNA.org) and analyzed the miRNA binding sites within the Tip110 3′UTR. We found a total of 14 miRNA binding sites with the Tip110 3′UTR (Figure 1A). The detailed seed sequence sites of the 14 miRNAs (11 different sequences including 3 repeats) were provided in Table 1. To determine the specificity of the miRNAs toward the Tip110 3′UTR, we transfected 293T with a Tip110 3′UTR Luc-reporter gene and the mimic of each miRNA (Table 2). Compared to the control, all these 11 miRNAs led to significant decreases of the Tip110 3′UTR-dependent luciferase reporter gene activities, with the most decreases for miR-300 and the least decreases with miR-193 (Figure 1B). Except for miR-214, all other miRNA led to decreases in Tip110 mRNA expression to various degrees (Figure 1C) and in parallel decreases in Tip110 protein expression (data not shown).

**Table 1:** Potential miRNA binding sequences(seed) for Tip110 3′UTR

Position 24–30 of Tip110 3′UTR Hsa-miR-124-3p.2	5′ ...CUGGGAGACAGGAAAUGCCUUAC... | | | | | | | 3' CGUAAGUGGCGCACGGAAUU
Position 69–75 of Tip110 3′UTR Hsa-miR-148b-3p	5′ ...CCCACCACCCAGCAGUGCACUGG... | | | | | | | 3′ UGUUUCAAGACACUACGUGACU
Position 82–88 of Tip110 3′UTR Hsa-miR-214-5p	5′ ...AGUGCACUGGGGAUGGACAGGCC... | | | | | | | 3' CGUGUCGUUCACAUCUGUCCGU
Position 1022–1028 of Tip110 3′UTR Hsa-miR-193a/b-3p	5′ ...AACAGCAACAGCAUUGGCCAGUU... | | | | | | | 3' UGACCCUGAAACAUCCGGUCAA
Position 633–639 of Tip110 3′UTR Hsa-miR-132-3p	5′ ...GUGCCACCUGCUGUGGACUGUUU... | | | | | | | | | | | | 3' GCUGGUACCGACAUCUGACAAU
Position 310–319 of Tip110 3′UTR Hsa-miR-494	5′ ...ACAGUUGUCC--AAAUGUUUCU... | | | : | | | | | | | | | 3′ CUCCAAAGGGCACAUACAAAGU
Position 550–572 of Tip110 3′UTR Hsa-miR-494	5′ ...GUCAGCCAUUGUUUCAUGUUUCC... : : | | | | | | | | | | 3′ CUCCAAAGGGCACA-UACAAAGU
Position 380–30 of Tip110 3′UTR Hsa-miR-186	5′ ...CACAUGUGCCCGUCAUUCUUUU... | | | | | | | 3′ UCGGGUUUUCCUCUUAAGAAAC
Position 931–951 of Tip110 3′UTR Hsa-miR-199a-5p	5′ ...UUAUA-GCAGUUUG-ACACUGGA... | : | | | | | : | | | | | | | | | 3′ CUUGUCCAUCAGACUUGUGACCC
Position 437–443 of Tip110 3′UTR Hsa-miR-219a-1-3p	5′ ...CUCAGUUCUAGCUGUUCAACUCU... | | | | | | | 3' GCCCUGCAGGUCUGAGUUGAGA
Position 443–449 of Tip110 3′UTR Hsa-miR-300	5′ ...UCUAGCUGUUCAACUCUUGUAUG... | | | | | | | 3' UCUCUCUCAGACGGGAACAUAU

**Table 2:** miRNA mimics sequences (seed sequences) used for targeting Tip110 3′UTR

miRNA mimic name	Sequence
hsa-miR-124-3p	UAAGGCACGCGGUGAAUGCC
hsa-miR-148b-3p	UCAGUGCAUCACAGAACUUUGU
hsa-miR-214-5p	UGCCUGUCUACACUUGCUGUGC
hsa-miR-494	UGAAACAUACACGGGAAACCUC
hsa-miR-186-5p	CAAAGAAUUCUCCUUUUGGGCU
hsa-miR-132-3p	UAACAGUCUACAGCCAUGGUCG
hsa-miR-338-3p	UCCAGCAUCAGUGAUUUUGUUG
hsa-miR-494	UGAAACAUACACGGGAAACCUC
hsa-miR-214-5p	UGCCUGUCUACACUUGCUGUGC
hsa-miR-199a-3p	ACAGUAGUCUGCACAUUGGUUA
hsa-miR-193a-3p	AACUGGCCUACAAAGUCCCAGU
hsa-miR-300	UAUACAAGGGCAGACUCUCUCU
hsa-miR-219-1-3p	AGAGUUGAGUCUGGACGUCCCG

### Human core blood CD34+ cell differentiation, Tip110 expression, and miRNA expression

**Figure 2:**
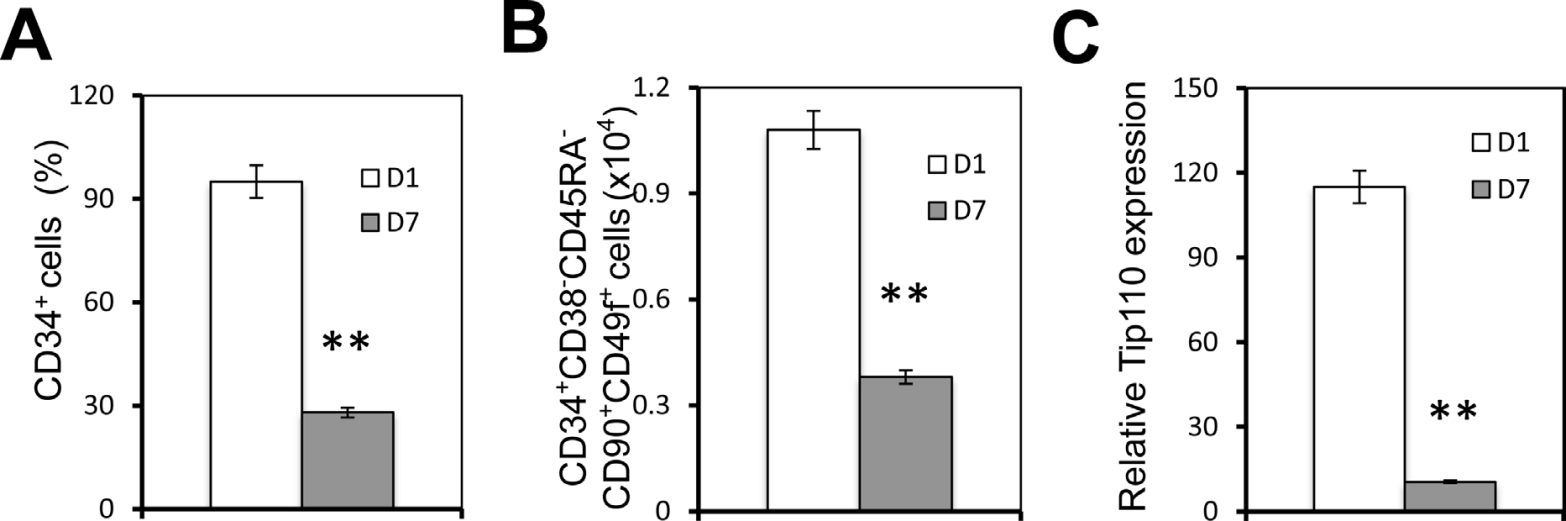
Human core blood CD34+ cell differentiation and Tip110 expression Human core blood CD34+ cells were isolated, cultured for 1 day (D1) or 7 days (D7), and stained followed by flow cytometry analysis for the CD34+ percentage (**A**) or CD34+CD38-CD45RA-CD90+CD49f+ cells (Y axis represents the number of cells out of 1 million cells) (**B**), or harvested for RNA isolation followed by qRT-PCR for Tip110 mRNA expression (**C**), which was calculated using β-actin as a reference. The data are the mean ± SEM of triplicates and are representative of three independent experiments.

**Figure 3:**
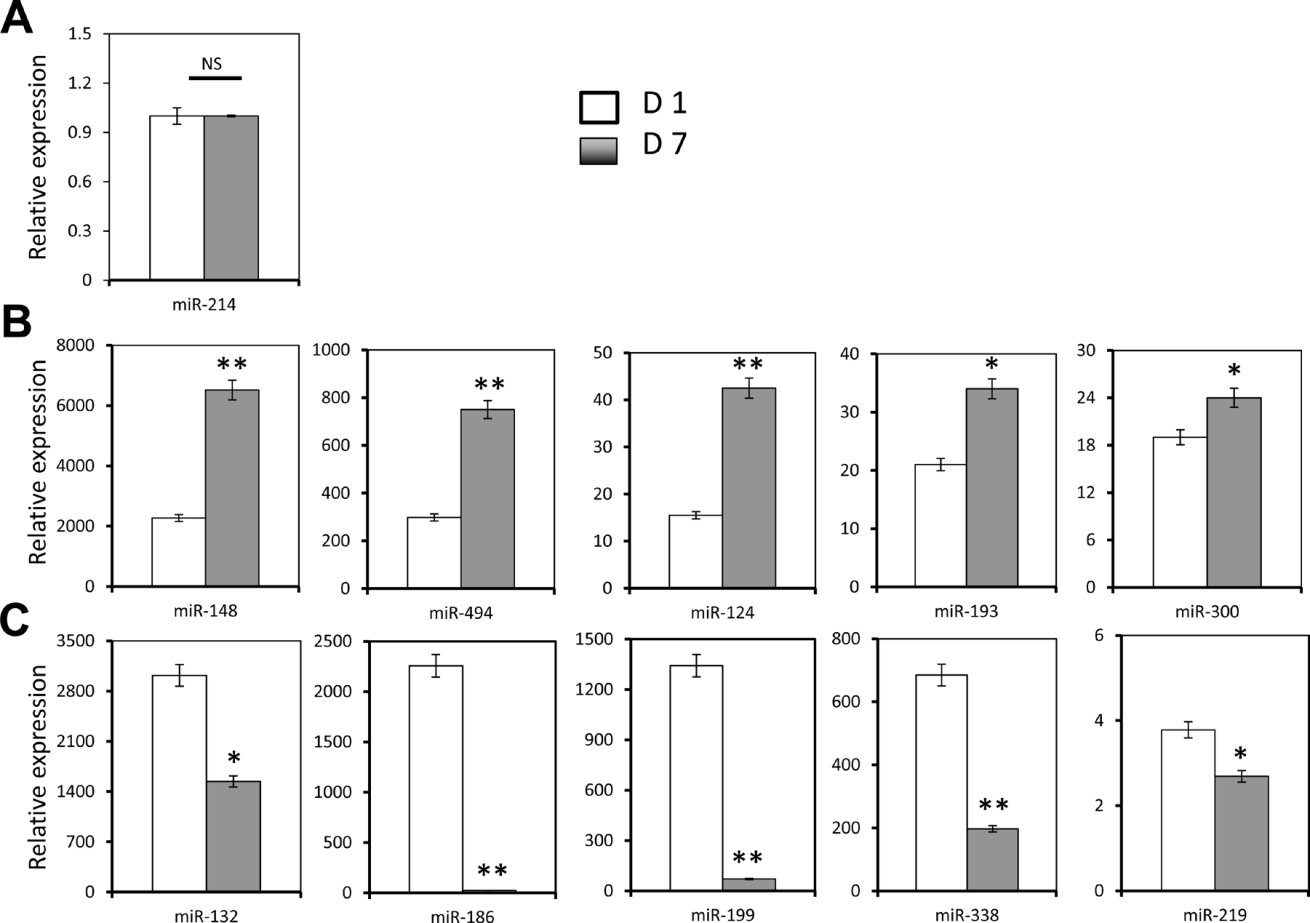
Effects of human core blood CD34+ cell differentiation on Tip110 miRNA expression Human core blood CD34+ cells were isolated, cultured for 1 day (D1) or 7 days (D7), and harvested for RNA isolation followed by qRT-PCR for miR-214 (**A**), miR-148, miR-494, miR-124, miR-193, and miR-300 (**B**), and miR-132, miR-186, miR-199, miR-338, and miR-219 (**C**). The data are mean ± SEM and are representative of three independent experiments.

We have previously shown that miR-124 is expressed in human core blood hematopoietic progenitor cells (HPCs) and it specifically binds to the Tip110 3′UTR and has a regulatory effect on core blood HPCs [7]. Next, we decided to evaluate expression of those identified miRNA during core blood CD34+ cell differentiation. We first determined the number of CD34+ cells and rigorously defined hematopoietic stem cells (HSC, CD34+CD38−CD45RA−CD90+CD49f+ cells) on day 1 and day 7 (CD34+ cells isolated from core blood counted as day 0). The purified cells were assessed for Tip110 expression by qRT-PCR. Core blood CD34+ cells cultured on days 1 and 7 were used to reflect undifferentiated and differentiating cells, respectively. The percentage of CD34+ cells was over 95% on day 1 and 30% on day 7 (Figure 2A). CD34+CD38−CD45RA−CD90+CD49f+ cells decreased by two thirds on day 7 compared to day 1 (Figure 2B). Tip110 was highly expressed on day 1 and decreased by 90% on day 7 (Figure 2C), which was consistent with our previous report that expression of Tip110 decreases as the cells differentiate [3]. We then determined the expression levels of 11 different miRNAs using this model. miR-214 showed the lowest expression level with no changes between day 1 and 7 (Figure 3A). We defined this expression as 1 (relative expression level calculation based on (C_T_) from qRT-PCR results). Expression levels of the other miRNAs were calculated as fold changes based on the miR-214 expression level of 1. miR-148, miR-494, miR-124, miR-193, and miR-300 showed increased expression levels from day 1 to 7. miR-148 showed very high expression levels (2272 to 6517 fold changes compared with that of miR-214) (Figure 3B), while miR-132, miR-186, miR-199, miR-338, and miR-219 showed decreased expression from day 1 to 7 (Figure 3C). Expression level changes suggest that these miRNAs may be involved in core blood CD34+ cell function and their differentiation.

### Tip110 3′UTR RNA expression led to increased Tip110 protein and mRNA expression levels

**Figure 4:**
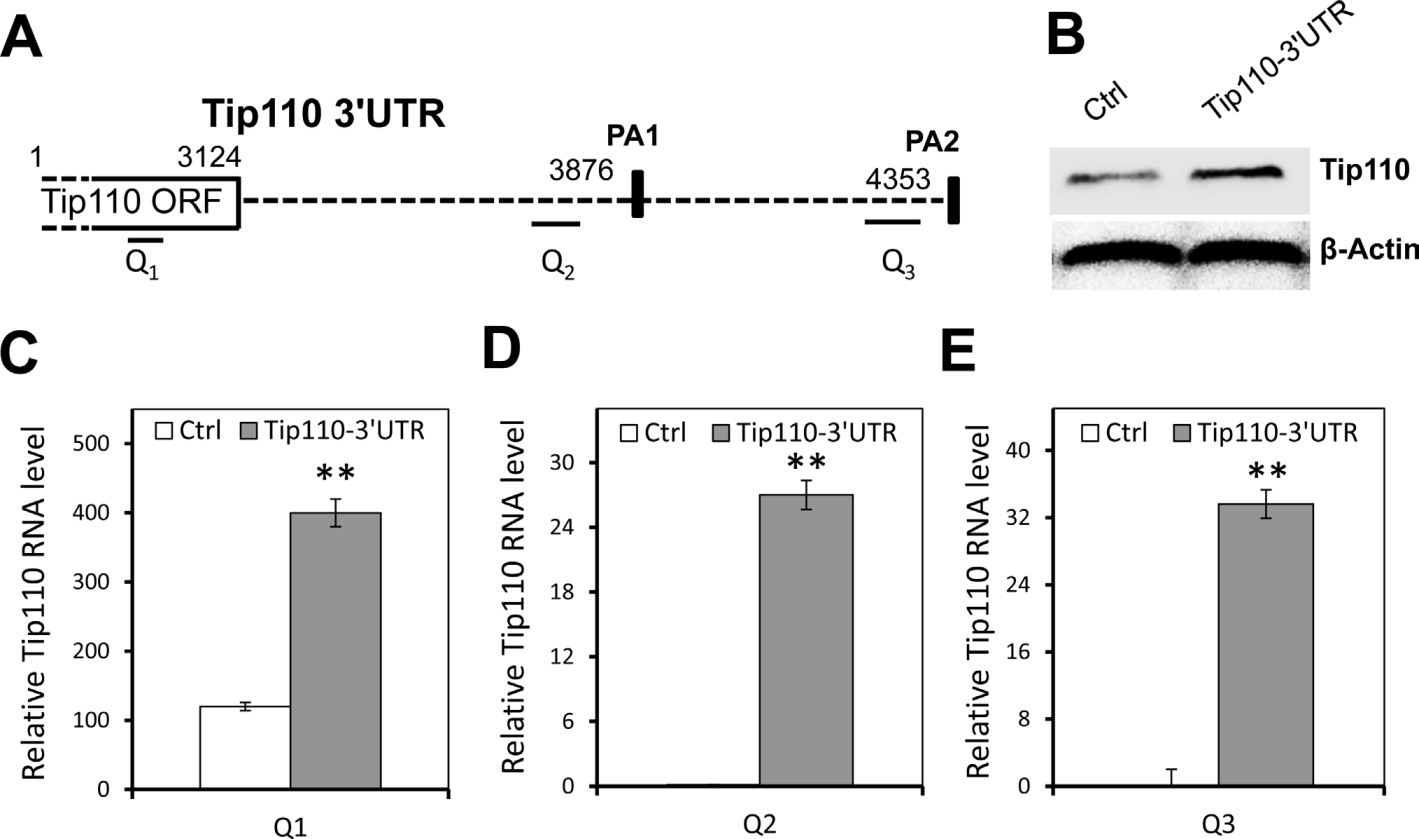
Up-regulation of constitutive Tip110 mRNA expression by expression of exogenous Tip110 3′UTR (**A**) Schematic of the Tip110 3′UTR region. The positions of p(A) sites (PA1 and PA2) are shown in solid rectangles. Q1, Q2 and Q3 are primer pairs specifically amplified Tip110 mRNA within the open reading frame (ORF), proximal 3′UTR, and distal 3′UTR, respectively. (**B**) 293T were transfected with a plasmid expressing the full-length Tip110 3′UTR RNA or the vector control, cultured for 48 hr, and harvested for cell lysates followed by Western blotting for Tip110 protein expression (B), or RNA isolation followed by qRT-PCR using primer pair Q1 (**C**), primer pair Q2 (**D**), or primer pair Q3 (**E**). β-actin was included as a loading control for both Western blotting and qRT-PCR. The data are expressed as mean ± SEM and are representative of three independent experiments.

Since the Tip110 3′UTR is a target of the above mentioned miRNAs, we took advantage of the effective competing endogenous RNA (ceRNA) strategy over the individual miRNAs [11–13]. Namely, we expressed Tip110 3′UTR RNA (Figure 4A) and used it as a decoy to compete with constitutive Tip110 3′UTR for any potential (identified and unidentified) Tip110 miRNAs. 293T were transfected with a plasmid expressing the full-length Tip110 3′UTR RNA or an empty vector control. Western blotting showed that Tip110 protein was expressed at least 3 times greater than the control (Figure 4B). We also determined the constitutive Tip110 mRNA expression using primers specifically targeted at different regions of the Tip110 mRNA transcripts (Table 3). Primer pair Q1 spanned the open reading frame of Tip110 and was used to determine the constitutive Tip110 mRNA expression. Primer pair Q2 spanned the first poly(A) site of the 3′UTR (PA1), and primer pair Q3 spanned the second poly(A) site of the 3′UTR (PA2). Both Q2 and Q3 were used to determine both the constitutive and transfected Tip110 3′UTR RNA expression. Consistent with constitutive Tip110 protein expression (Figure 4B), constitutive Tip110 mRNA from 3′UTR transfection was about 3 times greater than the control (Figure 4C). Compared to the control, the 3′UTR mRNA expression from the 3′UTR transfection was about 25–34 fold more than the constitutive Tip110 3′UTR mRNA using primer pairs Q2 and Q3 (Figure 4D and 4E). These findings confirm the feasibility of using the Tip110 3′UTR (other than individual Tip110 miRNA) to up-regulate Tip110 expression.

**Table 3:** Primers used in the study

Gene	GeneBank#	Region	Forward primer	Reverse primer	Expected size
Tip110	NM_014706	ORF: 1465-1629	Q1: CTTCATCCAGGCGACTGATT	Q1: TTGAAACGCTCTTCCACCTC	164 bp
		3′UTR: 3666-3899	Q2: GTAAATGACATGTCAGCCATTGTTTCATGT	Q2: GGCCACGCGTCGACTAGTAC	133 bp
		3′UTR: 4296-4399	Q3: TTGTGCATCAGTCGGGTTT	Q3: GGTAGGCACACAGGAGTTTATT	103 bp
β-Actin	NG_000840		AAACTGGAACGGTGAAGGTG	AGAGAAGTGGGGTGGCTTTT	174 bp

### Tip110 3′UTR RNA expression led to increased HSC and colony-forming units derived from human CB CD34+ cells

**Figure 5:**
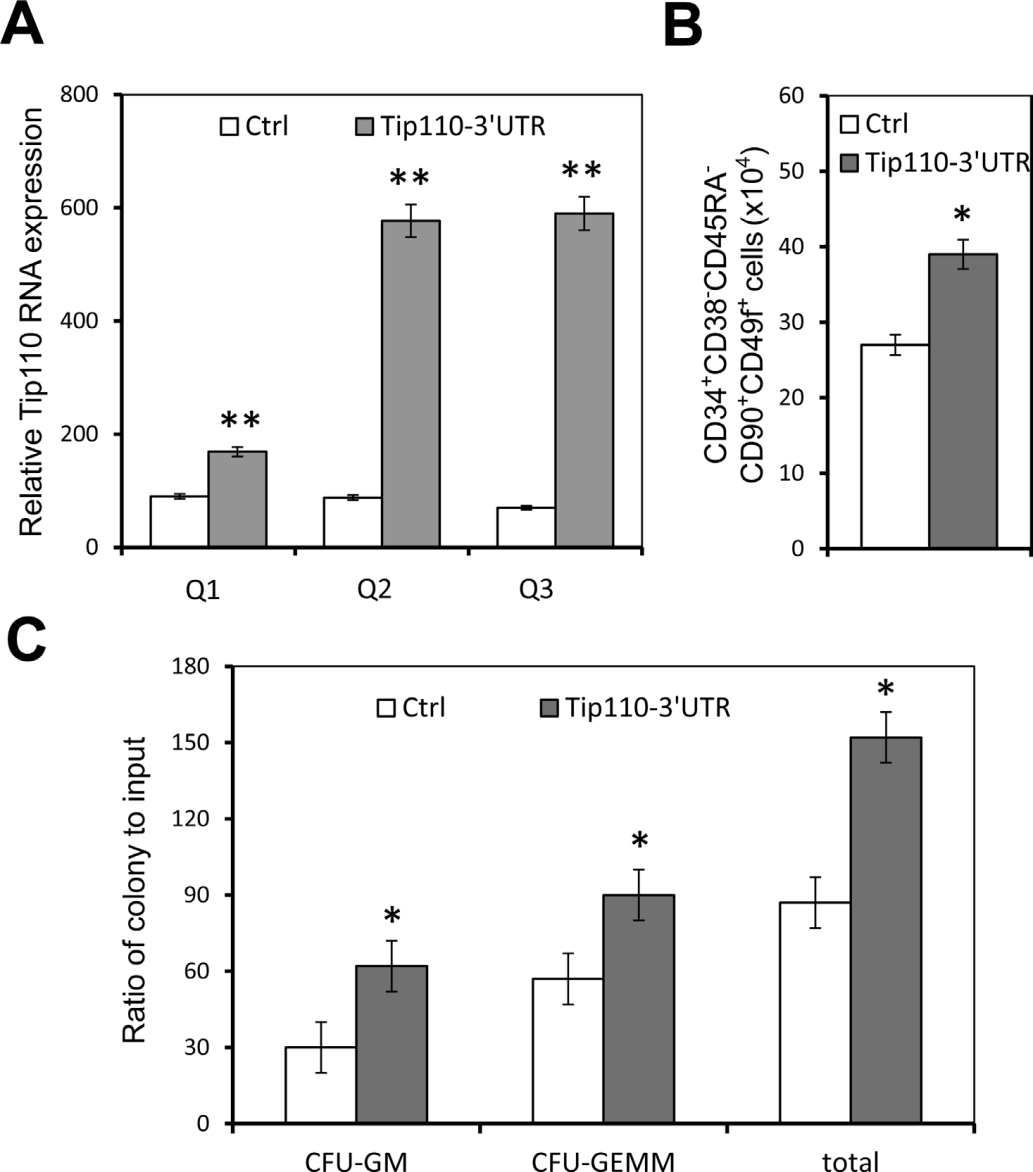
Effects of exogenous Tip110 3′UTR expression on human cord blood CD34 cell differentiation and hematopoietic colony formation Human cord blood CD34+ cells were isolated, cultured for one day, and transduced with lentiviruses expressing the full-length Tip110 3′UTR RNA. After culturing for 2 days, cells were harvested for RNA isolation followed by qRT-PCR for Tip110 expression using primer pair Q1, Q2 or Q3 (**A**), or for staining followed by flow cytometry analysis for CD34 cell differentiation into CD34+CD38^−^CD45RA^−^CD90^+^CD49f^+^ cells (**B**), or for hematopoietic colony formation assay (**C**). The data are mean ± SEM and are representative of three independent experiments.

To determine the potential roles of Tip110 miRNA in self-renewal and differentiation of human CD34+ hematopoietic cells, we next expressed the Tip110 3′UTR RNA in CB CD34+ cells. We transduced human CB CD34+ cells with lentiviruses expressing the Tip110 3′UTR RNA on day 1, and determined Tip110 expression as well as the number of rigorously defined HSCs on day 5. Similarly to the results with 293T, the Tip110 3′UTR RNA from the transduction was about 10 times higher compared to the control, assessed using both primer pairs Q2 and Q3 (Figure 5A). Meanwhile, constitutive Tip110 mRNA expression was increased about 2 fold resulting from the Tip110 3′UTR RNA expression, assessed using primer pair Q1 (Figure 5A). Tip110 3′UTR RNA expression in CB CD34+ cells also increased the number of rigorously defined human HSCs (Figure 5B). To assess whether the Tip110 3′UTR RNA expression would affect functionally assayable hematopoietic progenitor cells, a portion of these transduced CD34+ cells were subject to the colony assay. The cells transduced to express the Tip110 3′UTR RNA gave rise to about 2-fold increases in number of granulocyte macrophage (CFU-GM), multipotential (CFU-GEMM), and the total progenitors, compare to the control (Figure 5C). These results support the notion that Tip110 miRNA likely contributes to Tip110 expression, which in turn regulates self-renewal and differentiation of human CD34+ hematopoietic cells.

## DISCUSSION

**Figure 6:**
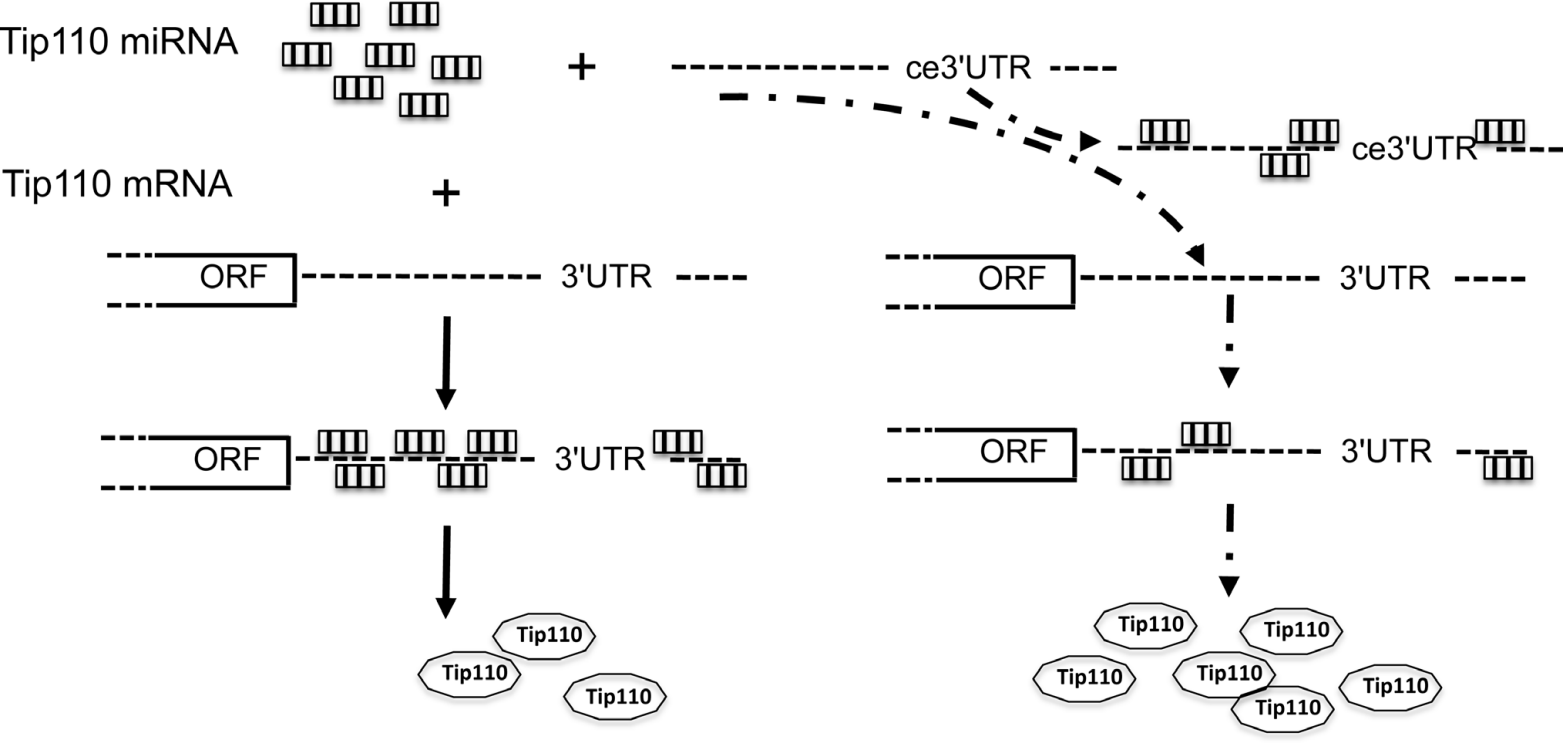
Schematic of Tip110 regulation mechanism through miRNAs and ceRNA Tip110 miRNAs bind to Tip110 3′UTR and lead to regulated Tip110 protein expression (left); In the presence of exogenous Tip110 3′UTR (ce3′UTR), ce3′UTR bind to Tip110 miRNAs, which leads to binding of less Tip110 miRNA to Tip110 3′UTR and increased Tip110 protein expression (right).

We identified more than 10 different miRNA binding sites on the Tip110 3′UTR. The specificity of these miRNAs for the 3′UTR of Tip110 was tested using a Tip110 3′UTR luciferase reporter gene assay. Those miRNAs were shown to be specific targets for the Tip110 3′UTR. Five miRNAs showed high levels of expression in core blood CD34+ cells and were down-regulated with differentiation; while 5 miRNAs showed low levels of expression in core blood CD34+ cells and were up-regulated with differentiation. To protect the integrity of the Tip110 3′UTR from degradation by miRNAs and to maintain Tip110 protein expression during the core blood CD34+ cell culture, we transduced human core blood CD34+ cells to express the Tip110 3′UTR RNA to compete the constitutive Tip110 3′UTR for Tip110 miRNAs. Ectopic expression of the Tip110 3′UTR RNA led to increased expression of constitutive Tip110 mRNA and protein expression (Figure 6). The relevance of our study is supported by our previous publications showing that up-regulation of Tip110 plays important roles in hematopoiesis [7, 8, 10].

From the analysis of the expression levels of these 11 miRNAs in core blood CD34+ cells cultured on day 1 vs. their differentiation on day 7, we grouped the miRNA expression pattern into three categories. The first showed no expression changes from day 1 to day 7, and included only miR-214. Its expression level was the lowest confirmed to this point. The second group that had a low expression level on day 1 and a high expression level on day 7 included miR-148, miR-494, miR-124, miR-193, and miR-300. This group of miRNAs might be important for regulating genes that are highly expressed in the undifferentiated state, such as Tip110, which we identified here, as well as other potential genes. The third category had a high expression level on day 1 and down-regulated expression on day 7, and included miR-132, miR-186, miR-199, miR-338, and miR-219. This group of miRNAs might be important for regulating genes that are highly expressed in the differentiating state. miRNA regulation of gene expression is very complicated in that each microRNA regulate thousands of mRNA and each mRNA is regulated by many microRNAs [14, 15]. Tip110 expression level is down-regulated in differentiating CD34+ cells. It is reasonable to assume that Tip110 down-regulation during CD34+ cell differentiation is a combined effect of increased microRNA levels targeting 3′UTR of Tip110 to down regulate its expression over decreased microRNAs of alleviation targeting 3′UTR of Tip110 in differentiating CD34+ cells.

Competing endogenous RNA (ceRNA) networks basically regulate all biological process [11–13]. We hypothesized that expressing non-coding RNA transcripts of the Tip110 3′UTR may have a dramatic function in regulating Tip110 gene expression, an hypothesis substantiated when we introduced the 3′UTR RNA to core blood CD34+ cells to up-regulate Tip110 expression. Since transduction/transfection efficiency in 293T cells is much higher compared to core blood CD34+ cells, the constitutive Tip110 expression was higher than that of core blood CD34+ cells when expressing the Tip110 3′UTR RNA (Figures 4 and 5). Our results thus suggest that ectopically expressed Tip110 3′UTR RNA acts to compete with constitutive Tip110 3′UTR RNA for miRNAs binding, resulting in up-regulated expression of Tip110, so as to influence HSCs and their differentiation. Expression of the 3′UTR may have greater advantage in modulating cell activities compared with miRNA inhibitors, in that the 3′UTR has the capacity to modulate multiple miRNAs (identified and unidentified), providing a potential use for 3′UTR transcripts. Further mechanistic understanding of Tip110 regulation through miRNAs and ceRNA might help us identify novel ways to enhance the function of core blood HSCs for expansion and clinical transplantation.

## MATERIALS AND METHODS

### Plasmids

pCSC.GFP was described previously [8]. pLighSwitch.Luc-Tip110-3′UTR was purchased from SwitchGear Genomics (Carisbad, CA, USA) and contained the full-length Tip110 3′UTR (1346 nt). CSC-Tip110-3′UTR was constructed to express Tip110 3′UTR RNA by cloning the Tip110 3′UTR into pCSC.GFP.

### miRNA analysis

For each RNA sample, 500 ng RNA was used to prepare cDNA using a qScript microRNA cDNA Synthesis Kit (Quanta PN 95107). After cDNA synthesis, an equivalent of 10 ng the original RNA sample was mixed with Perfecta SYBR Green SuperMix (Quanta PN 95054) and Universal PCR Primer (Quanta PN 95109) with each PerfeCta microRNA Assay Primer (miR-193a-3p, miR-300, miR-186-5p, miR-494, miR-199a-5p, miR-210-1-3p, miR-338-3p, miR-132a-3p, miR-148a-3p, and miR-124a-3p) in 10 μl qPCR reactions. The qPCR plates were run in a CFX96 real-time PCR detection system (Bio-Rad) using a two-step cycling protocol (95°C for 2 min followed by 40 cycles of 95°C for 10 s and 60°C for 30 s) and followed by a melting curve. Threshold cycle (CT) values were calculated using Bio-Rad CFX manager software. 2^(-DDCT)^ value was calculated to represent the fold change of the target gene mRNA.

### Cells, cell transfection, and luciferase reporter gene assay

293T were purchased from ATCC. Human core blood was collected and used as reported [8, 9]. CD34^+^ cells were purified within 24 hr of collection using immunomagnetic selection (Miltenyi Biotec). CD34+ cells (>93% pure) were cultured in IMDM containing 1% or 10% FBS with 100 ng/mL of human stem cell factor (SCF), 100 ng/mL human FLT3 ligand (FL), and 100 ng/mL of human thrombopoietin (Tpo) from R&D Systems (Minneapolis, MN, USA). Cell transfections were performed on the Nucleofector device (Amaxa Biosystem) according to the manufacturer's instructions [16]. pcDNA3 was used to equalize amounts of DNA transfected for all transfections. Luciferase reporter gene activities were determined using the Firefly/Renilla luciferase system (Promega, Madison, WI, USA). pTKβGal was included to normalize variations in transfection efficiency for all transfections. miRNA transfections used Lipofectamine 2000 LTX (Invitrogen).

### Preparation of whole cell lysates and Western blotting

Cells were washed twice with ice-cold PBS and then harvested for whole cell lysates [17]. Briefly, cell pellets were suspended in two volumes of whole cell lysis buffer (10 mM NaHPO_4_, 150 mM NaCl, 1% Triton X-100, 0.1% SDS, 0.2% sodium azide, 0.5% sodium deoxycholate, 0.004% sodium fluoride, and 1 mM sodium orthovanadate), and incubated on ice for 10 min. Whole cell lysates were obtained by centrifugation and removal of the cell debris. For Western blotting, cell lysates were electrophoretically separated on 10% SDS-PAGE and analyzed by immunoblotting using an anti-Tip110 antibody [8] and appropriate horseradish peroxidase-conjugated secondary antibodies, followed by visualization with the ECL system (GE Healthcare Bio-sciences, Pittsburgh, PA, USA).

RNA isolation and qRT-PCR Total RNA was extracted using TRizol (Life Technology, Grand Island, NY, USA) according to the manufacturer's instructions. cDNA was synthesized using a ScriptII RT Reagent Kit (Promega). cDNAs were used for qPCR performed by Power SYBR® Green PCR kit (Life Technologies, Grand Island, NY, USA) according to the manufacturer's instructions. Primers for Tip110 and actin were described previously [18]. The qPCR program consisted of 1 cycle of 95°C for 10 min; 40 cycles of 95°C for 15 sec and 60°C for 1 min. Bio-Rad CFX manager software was used for calculating gross-threshold (C_T_) values. The 2^(-ΔΔCT)^ was calculated to represent the fold change of gene expressions. mRNA were normalized using β-actin as the reference.

### Flow cytometry analysis of human core blood CD34+ cells

Freshly purified and cultured human core blood CD34+ cells from the different experimental groups were washed twice with PBS and stained with mouse anti-human CD34-PE/CD34-APC/CD34-FITC antibody, mouse antihuman CD38-PE antibody, mouse anti-human CD45RA-PECF594, mouse anti-human CD90-PECy7, and mouse anti-human CD49f-PerCPCy5.5 in the dark at room temperature for 30 min. All antibodies were from BD Biosciences (San Jose, CA, USA). Mouse IgG-FITC, mouse IgGAPC, and mouse IgG-PE were included as negative controls. Cells were subjected to FACS flow cytometric analysis (Becton Dickinson).

### Lentivirus transduction and *in vitro* hematopoietic colony-forming cell assay

Tip110-3′UTR.GFP lentivirus preparation and transduction were described previously [7]. Human core blood CD34+ cells were seeded in triplicate onto 35 mm dishes at a density of 250 cells per dish in 1.0 ml of 1% methylcellulose culture medium containing 100 mM 2-mercaptoethanl, 2 mM L-glutamine, 30% fetal bovine serum (FBS; Hyclone Laboratories, Logan, UT, USA). Recombinant human erythropoietin (1 U/ml), SCF (50 ng/ml), human interleukin-3 (IL-3; 10 ng/ml), and granulocyte macrophage colony stimulating factor (GM-CSF; 10 ng/ml) were included in the culture medium to stimulate colonies from multipotential (CFU-GEMM), and granulocyte macrophage (CFU-GM) progenitor cells. Epo was purchased from Amgen Inc. (Thousand Oaks, CA, USA). IL-3 and GM-CSF, were purchased from R & D Systems (Minneapolis, MN, USA). Colonies were scored after 14 days incubation at 5% CO_2_ and lowered (5%) O_2_ tension [19].

### Statistical analysis

Data are mean ± SD, unless otherwise noted and are representative of at least three independent experiments. All experimental data were analyzed by 2-tailed student's *t*-test.
